# Natural Killer Cells Prevent the Formation of Teratomas Derived From Human Induced Pluripotent Stem Cells

**DOI:** 10.3389/fimmu.2019.02580

**Published:** 2019-11-07

**Authors:** Basma Benabdallah, Cynthia Désaulniers-Langevin, Chloé Colas, Yuanyi Li, Guy Rousseau, Jean V. Guimond, Elie Haddad, Christian Beauséjour

**Affiliations:** ^1^Centre de recherche du CHU Sainte-Justine, Montreal, QC, Canada; ^2^Centre de recherche du CIUSSS du Nord-de-Île-de Montréal, Montreal, QC, Canada; ^3^Département de Pharmacologie et Physiologie, Faculté de Médecine, Université de Montréal, Montreal, QC, Canada; ^4^CIUSSS du Centre-Sud-de-l'Ile-de-Montréal, Montreal, QC, Canada; ^5^Département de Pédiatrie, Faculté de Médecine, Université de Montréal, Montreal, QC, Canada

**Keywords:** induced pluripotent stem cell, humanized mice, adoptive transfer, NK cells, T cells

## Abstract

The safe utilization of induced pluripotent stem cell (iPSC) derivatives in clinical use is attributed to the complete elimination of the risk of forming teratomas after transplantation. The extent by which such a risk exists in immune-competent hosts is mostly unknown. Here, using humanized mice reconstituted with fetal hematopoietic stem cells and autologous thymus tissue (bone–liver–thymus humanized mice [Hu-BLT]) or following the adoptive transfer of peripheral blood mononuclear cells(PBMCs) (Hu-AT), we evaluated the capacity of immune cells to prevent or eliminate teratomas derived from human iPSCs (hiPSCs). Our results showed that the injection of hiPSCs failed to form teratomas in Hu-AT mice reconstituted with allogeneic or autologous PBMCs or purified natural killer (NK) cells alone. However, teratomas were observed in Hu-AT mice reconstituted with autologous PBMCs depleted from NK cells. In line with these results, Hu-BLT, which do not have functional NK cells, could not prevent the growth of teratomas. Finally, we found that established teratomas were not targeted by NK cells and instead were efficiently rejected by allogeneic but not autologous T cells in Hu-AT mice. Overall, our findings suggest that autologous hiPSC-derived therapies are unlikely to form teratomas in the presence of NK cells.

## Introduction

The potential of regenerative medicine is greatly enhanced by the development of induced pluripotent stem cells (iPSCs). Yet the risk of forming teratomas using iPSC-derived cell grafts could compromise their clinical use. Indeed, the injection of only a few hundred human embryonic cells was sufficient to form a teratoma in immunodeficient mice (1). To prevent the formation of teratomas, small molecules able to selectively and efficiently kill pluripotent cell by inhibiting antiapoptotic factors were developed (2, 3). However, the need for pluripotent stem cells to evade immune responses may be required for the growth of teratomas *in vivo*. Indeed, Zhao et al. were the first to observe that mouse iPSCs, but not embryonic stem cells (ESCs), are immunogenic and fail to form teratomas when injected subcutaneously in syngeneic recipients (4). A response they showed was dependent on the abnormal overexpression of immunogenic genes in iPSCs and on the presence of antigen-presenting cells at the injection site (4, 5). However, the pioneering work by Dressel et al. showed that mouse iPSCs can give rise to teratomas in autologous/syngeneic mice in the absence of the activation of the host natural killer (NK) cells (6, 7). Likewise, human iPSCs (hiPSCs) were shown to be the target of human NK cells *in vitro* (8, 9). Yet the *in vivo* contribution of the innate immunity, particularly the role of NK cells on the tumorigenic potential of hiPSCs remains unknown. Here, we used two different models of humanized mice: (i) Hu-bone–liver–thymus (BLT) mice generated by the co-transplantation of fetal liver hematopoietic stem cells along with autologous human thymus tissues that allow for the development and maturation of competent human T cells and (ii) Hu-AT mice reconstituted following the adoptive transfer (AT) of adult peripheral blood mononuclear cells (PBMCs); and we demonstrated that teratoma formation by hiPSCs is abolished only in the presence of NK cells and that this NK-specific cytotoxicity is lost upon the differentiation of hiPSCs.

## Experimental Procedures

### Humanized Mice

NOD/SCID/IL2Rγ^null^ (NSG) mice were obtained from the Jackson Laboratory (Bar Harbor, ME) and housed in the animal care facility at the CHU Sainte-Justine Research Center under pathogen-free conditions in sterile ventilated racks. All *in vivo* manipulations were previously approved by the institutional committee for good laboratory practices for animal research (protocol #579). Bone–liver–thymus humanized mice (Hu-BLT) were generated as previously described (10). Briefly, 6-week-old NSG mice were first irradiated with 2 Gy of total body irradiation (1 Gy/min using a Faxitron CP-160) and implanted with small pieces (1–2 mm^3^) of human fetal thymus under the renal capsule followed by the intravenous delivery of 1 × 10^7^ CD34^+^ hematopoietic stem cells isolated from autologous fetal liver. Fetal tissues were obtained from consented healthy donors after surgical abortion at around week 20 of pregnancy. Human immune cell engraftment in humanized mice was monitored in peripheral blood until 13 weeks post-reconstitution. Leukocytes were labeled with conjugated antibodies for human PerCP-Cy5.5-CD45, APC-CD3, PE-CD19, and FITC-CD4 (see Table 1 in the Supplementary File for a complete list of antibodies used) and analyzed by flow cytometry (BD FACSCANTO II, BD Biosciences). For AT experiments (Hu-AT), human adult blood was collected and immune cells were purified by Ficoll (GE Healthcare). Mice were injected intravenously with 1 × 10^7^ freshly isolated PBMCs or NK-depleted PBMCs obtained from the negative fraction of a positive selection (CD56^+^) kit (catalog #17855 from STEMCELL Technologies). Alternatively, mice were injected with 5–15 × 105 NK cells purified using the NK-cell enrichment negative selection kit (catalog #19055 from STEMCELL Technologies).

### Generation and Characterization of Human Induced Pluripotent Stem Cells

PBMCs or fibroblasts obtained either from human fetal liver tissues or human adult skin were isolated after collagenase dissociation and reprogrammed into iPSCs with the integration-free based Sendai virus (Cytotune 2.0 kit catalog #A16517 from Life Technologies). Fibroblasts were used at low population doubling (<5) to insure high efficiency of reprogramming. Emerging hiPSC colonies were manually picked and cultured under feeder-free conditions in Essential 8 medium on Geltrex-coated dishes (Life Technologies). hiPSC clones were maintained in Essential 8 Flex medium (Life Technologies) in feeder-free conditions and passaged at least 15 times to increase stable pluripotency. hiPSC generation and characterization were performed in the iPSC cell reprogramming core facility of CHU Sainte-Justine. hiPSC colonies were stained with antibodies for anti-human SSEA-4, Sox2, OCT4, and TRA1-60 followed by incubation with appropriate ALEXA-conjugated secondary antibodies using the pluripotent Stem Cell 4-Marker Immunocytochemistry Kit following the manufacturer's instructions (catalog #A24881 from Life Technologies). Karyotypes were produced by G-banding and analyzed by the CHU Sainte-Justine Cytogenetic Department.

### Flow Cytometry-Based Phenotypic Characterization

Parental fibroblasts and their derived hiPSCs colonies were cultured in Essential 8 Flex medium and dissociated in a single-cell suspension using TrypLE (Life Technologies) and characterized by flow cytometry (BD LSRFortessa, BD Biosciences) for their expression of the human leukocyte antigen-I (HLA-I), the three common costimulatory molecules (CD80, CD83, and CD86), and ligands for NK-cell stimulatory receptors (MICA/B and CD112/CD155; see Table 1 in the Supplementary File for a complete list of antibodies used).

### T-Cell Activation and Proliferation Assays

Human PBMCs were freshly isolated from healthy donors' peripheral blood using Ficoll-Paque. Effector cells were then cocultured with either autologous or allogeneic iPSCs at 1:2 ratio during 3 days for T-cell activation or 5 days for T-cell proliferation at 37°C. T-cell activation was measured with a phycoerythrin (PE)-conjugated anti-hCD69 (Biolegend) on CD3^+^-gated viable cells by flow cytometry (BD LSRFortessa, BD Biosciences). For T-cell proliferation, PBMCs were first stained using the CellTrace CFSE kit to monitor distinct generations of viable proliferating CD3^+^ T cells by dye dilution (catalog #C34570 from Invitrogen) before being cocultured with iPSCs. Effector cells without stimulation were used as a negative control, and an anti-CD3 (OKT3) (Biolegend) antibody was used as a positive control. 7-AAD (catalog #51.68981E from BD Biosciences) was used to exclude dead cells.

### Natural Killer Cell Degranulation and Cytotoxicity Assays

Freshly purified NK cells (as described above) were incubated with or without target cells at the indicated ratios. K562 cells were used as a positive control in all experiments. For the NK-cell degranulation assay, effector, and target cells were cocultured at 1:2 ratio in the presence of fluorescein isothiocyanate (FITC)-conjugated anti-human CD107a/b (BD Biosciences) for 1 h at 37°C, and then 2 μl/ml of monensin (catalog #554724 from BD Biosciences) was added to the cell mixture for an additional 3 h of incubation. For cytotoxicity assay, effector, and PKH26-stained target cells were mixed at 1:1 or 5:1 ratio and incubated for 4 h at 37°C. At the end of the incubation, degranulation was quantified by flow cytometry (BD LSRFortessa, BD Biosciences) after gating on CD3^−^/CD56^+^/CD107^+^ viable cells, and the extent of cytotoxicity was determined by the relative number of live target cells labeled with PKH26 only and dead cells labeled with both PKH26 (catalog #PKH26GL-1KT from Sigma-Aldrich) and 7-AAD (BD Biosciences).

### Teratoma Formation

Approximately 1 × 10^6^ hiPSCs were first detached in cell clumps using the gentle cell dissociation reagent (STEMCELL Technologies) and resuspended in cold phosphate-buffered saline (PBS) containing 50% of Geltrex and kept on ice. Cell mixture was then injected unilaterally under the renal capsule of mice. Eight weeks later, teratomas were dissected out and placed in OCT. Cryosections were stained with hematoxylin and eosin for histological analysis or were immunostained with antibodies against human CD4, CD8 (Biolegend), NKp46 (R&D Systems), and TUNEL (catalog #1684795910 from Roche). Immune cell infiltration scores were collected by two blinded evaluators. The average number of cells present in five different fields in each condition was attributed + for a low level of infiltration (defined as <10 cells/field) and ++++ for a high level of infiltration (more than 100 cells/field). Of note, although all iPSC clones used in this study were validated to form teratomas *in vivo*, not all individual tumors/teratomas were analyzed histologically.

### Statistical Analysis

GraphPad Prism 8 software was used for statistical analysis; *p*-values on multiple comparisons were calculated using Student's *t*-test. Alternatively, a chi-square analysis was performed to determine if the presence of teratoma formation was significantly different between conditions. First, we have performed an overall chi-square analysis followed, when *p* < 0.05, by a two-by-two comparison for which a *p*-value was adjusted using Bonferroni correction for the number of comparisons. The analyses were performed using SPSS (version 25), and *p* < 0.05 was considered significant.

## Results

### Natural Killer Cells Prevent the Formation of Human Induced Pluripotent Stem Cell-Derived Teratomas in Hu-AT Mice

**Figure 1:**
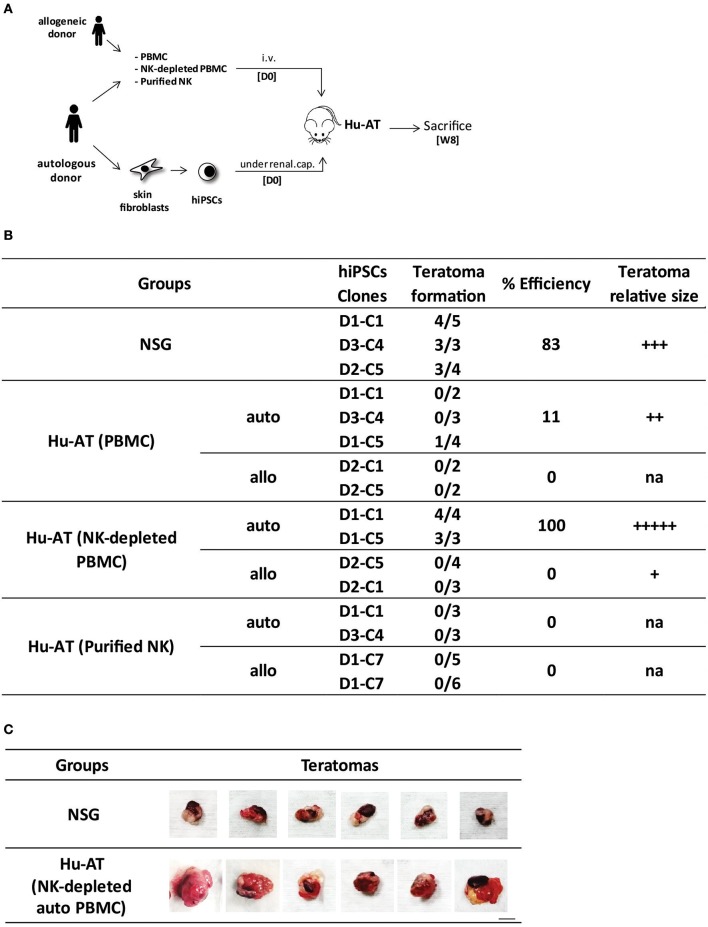
Injection of NK cells is sufficient to prevent the formation of hiPSC-derived teratomas in Hu-AT mice. **(A)** Experimental design and timeline of the injection of hiPSCs in Hu-AT mice. PBMCs (1 × 10^7^), NK-depleted PBMCs (1 × 10^7^), or purified NK cells (5–15 × 10^5^) were injected intravenously at day 0 (DO) to generate Hu-AT mice. Approximately 1 × 10^6^ autologous or allogeneic hiPSCs was injected under the renal capsule on the same day (one injection per mouse). Teratoma formation was evaluated upon sacrifice 8 weeks after the injection of cells. **(B)** Incidence of teratoma formation in the renal capsule of Hu-AT mice. Shown is the proportion of teratomas derived from the indicated hiPSC clones. A significant overall difference was observed between Hu-AT (NK-depleted PBMCs) autologous and allogeneic, χ^2^(6) = 44.28, *p* < 0.05. Of note, allogeneic D1-C7 is listed twice as it was used in Hu-AT mice from two independent experiments using NK cells from two different donors. **(C)** Representative photos showing the increased size of teratomas isolated from NSG mice previously injected with NK-depleted autologous PBMCs compared with control NSG non-reconstituted mice. Scale bar, 1 cm. NK, natural killer; hiPSC, human induced pluripotent stem cell; PBMCs, peripheral blood mononuclear cell; NSG, NOD/SCID/IL2Rγ^null^.

To investigate if immune cells could protect against the formation of hiPSC-derived teratomas, we first generated hiPSC clones from skin fibroblasts or PBMCs collected from different donors (see Table 2 in the Supplementary File) using an integration-free (Sendai virus) approach. We confirmed that these clones had normal karyotypes, expressed the classical markers of pluripotency, and were able to form the three germ layers in NSG immunodeficient mice (Figures S1A,B and data not shown). We then injected various hiPSC clones under the renal capsule of NSG mice and determined if the AT of 1 × 10^7^ PBMCs could prevent the formation of teratomas (Figure 1A). Our results show that practically no teratomas were found (1/13) after the AT of autologous or allogeneic PBMCs (Figure 1B). In comparison, 10/12 teratomas formed in the absence of the injection of PBMCs. To better define the role of T and NK cells in this process, we repeated the experiment using either NK-depleted PBMCs or purified NK cells (Figure S2). We found that hiPSCs efficiently formed teratomas (7/7) in the presence of autologous PBMCs depleted from NK cells. This was in contrast to when mice were injected with allogeneic NK-depleted PBMCs, in which case teratomas were rejected (0/7). A significant overall difference was observed between the different conditions, χ^2^(6) = 44.28, *p* < 0.05. Further analysis reveals a significant difference between NSG and all Hu-AT conditions (*p* < 0.05) and between Hu-AT (NK-depleted PBMCs) autologous and allogeneic (*p* < 0.05). No other significant difference was detected. This was because in the absence of NK cells, hiPSCs were allowed to grow into teratomas and that differentiated cells were then rejected by allogeneic T cells. Intriguingly, we observed that mice injected with NK-depleted PBMCs had larger teratomas compared with those grown in immunodeficient mice (Figure 1C). We speculate that their enhanced growth was fueled by human cytokines secreted by autologous PBMCs. Importantly, our results also show that the injection of purified NK cells (5–15 × 10^5^) was able to prevent the formation of hiPSC-derived teratomas (0/17) in both autologous and allogeneic settings (Figure 1B). This confirms that NK cells, but not autologous T cells, are efficient in preventing the development of hiPSC-derived teratomas.

### Human Induced Pluripotent Stem Cells Are Targeted by Natural Killer but Not T Cells *in vitro*

**Figure 2:**
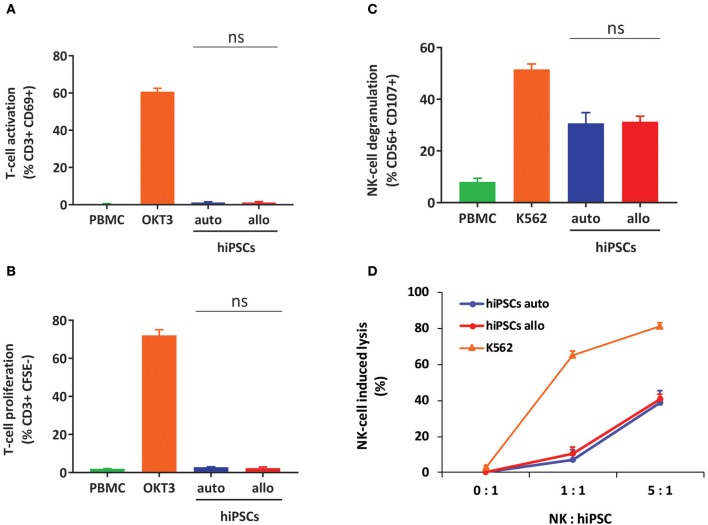
hiPSCs are the target of NK but not T cells *in vitro*. **(A)** Measures of T-cell activation (as determined by gating for CD3^+^/CD69^+^ cells) after coculture of hiPSCs with autologous or allogeneic PBMCs for 3 days. PBMCs alone were used as a negative control, and PBMCs treated with OKT3 were used as a positive control. Shown is the mean ± SEM of *n* = 4 from two independent experiments using effector cells collected from two different donors each time. **(B)** Measures of T-cell proliferation (as determined by gating for CD3^+^/CFSE^+^ cells) after coculture of hiPSCs with autologous or allogeneic CFSE-stained PBMCs for 5 days. PBMCs alone were used as a negative control, and PBMCs treated with OKT3 were used as a positive control. Shown is the mean ± SEM of *n* = 4 from two independent experiments using effector cells collected from two different donors each time. **(C)** hiPSCs induce degranulation of NK cells *in vitro*. Degranulation was determined by evaluating CD107 expression on gated CD3^−^/CD56^+^ NK-cell populations after a 4-h coculture between freshly isolated PBMCs and autologous or allogeneic hiPSCs (ratio 1:2). PBMCs alone were used as a negative control, and PBMCs cocultured with K562 cells were used as a positive control. Shown is the mean ± SEM of *n* = 4 from two independent experiments using effector cells collected from two different donors each time. **(D)** Autologous and allogeneic hiPSCs are lysed by purified NK cells *in vitro*. Cell lysis was determined by flow cytometry with the absolute count of PKH26-stained hiPSCs after a 4-h coculture with NK cells purified by magnetic negative selection. NK cells alone were used as a negative control, and NK cells cocultured with K562 cells were used as a positive control. Shown is the mean ± SEM of *n* = 4. hiPSCs, human induced pluripotent stem cells; NK, natural killer; PBMCs, peripheral blood mononuclear cell; CFSE, carboxyfluorescein succinimidyl ester.

To confirm our results on the cytotoxicity of T and NK cells toward hiPSCs in Hu-AT mice, we next performed *in vitro* assays of T- and NK-cell responses against autologous or allogeneic hiPSCs. We observed that although hiPSCs were the target of NK cells, they were unable to activate T cells (Figure 2). This suggests that hiPSCs are prone to NK but not to T cell killing, an effect that could be mediated by the presence of NK activating receptors and low expression of HLA-I and costimulatory molecules at the surface of hiPSCs (Figure S1C).

### Human Induced Pluripotent Stem Cells Efficiently Form Teratomas in Hu-BLT Mice Lacking Functional Natural Killer Cells

**Figure 3:**
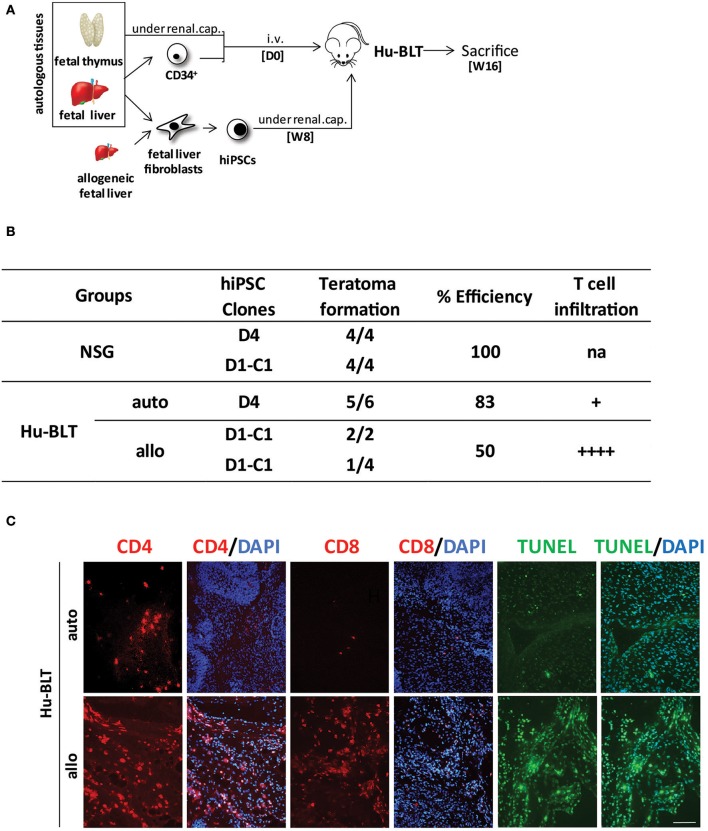
hiPSCs efficiently develop into teratomas in Hu-BLT mice lacking functional NK cells. **(A)** Experimental design and timeline of the injection of hiPSCs in Hu-BLT mice. Hu-BLT mice were generated by the injection of fetal tissues as previously described and immune reconstitution allowed for 8 weeks (W8) prior from the injection of ~1 × 10^6^ hiPSCs under the renal capsule. hiPSCs were derived from fetal liver fibroblasts autologous or allogeneic to CD34^+^ hematopoietic cells. Teratomas were allowed to grow for 8 weeks before mice were sacrificed at week 16 (W16). **(B)** Incidence of teratoma formation in the renal capsule of Hu-BLT mice. Shown is the proportion of teratomas derived from the indicated hiPSC clones and their relative infiltration with T cells. No significant difference was observed between conditions (χ^2^(2) = 5.42, *p* = 0.067) in spite of a clear trend. Of note, allogeneic D1-C1 is listed twice as it was used in Hu-BLT mice reconstituted with cells and thymus tissue obtained from two different donors. na indicates not applicable, + indicates a low level of infiltration (<10 cells/field on average), and + + ++ indicates a high level of infiltration (more than 100 cells/field on average). **(C)** Representative photos showing the infiltration of CD4^+^ and CD8^+^ T cells (in red) and apoptosis (as determined by TUNEL in green) in sections of allogeneic and autologous teratomas collected from Hu-BLT mice. DAPI staining was performed to visualize nuclei (in blue). Showed are photos taken at 20×. Scale bar, 100 μm. hiPSCs, human induced pluripotent stem cells; NK, natural killer.

To further investigate the role of NK cells in the development of hiPSC-derived teratomas, we then injected hiPSCs under the renal capsule of fully reconstituted Hu-BLT mice generated after the transplantation of fetal CD34 cells and thymus tissue fragments (Figure 3A). Hu-BLT mice are known to support reconstitution, maturation, and selection of T cells but lack functional NK cells (Figure S3) (11). Consistent with our findings in Hu-AT mice, our results show that the growth of autologous hiPSC-derived teratomas was not significantly hampered in Hu-BLT mice (8/8 in NSG mice vs. 8/12 in Hu-BLT mice, see Figure 3B). However, teratomas formed in Hu-BLT mice (5/6) injected with autologous immune cells were found infiltrated with low-level, non-randomly distributed T cells with no significant sign of apoptosis (Figure 3C), supporting the findings of Zhao et al. (12). Out of the five teratomas that formed in autologous Hu-BLT mice, only one was bigger in size when compared with teratomas formed in NSG mice (data not shown). This oversized teratoma was observed in the mouse that had the highest level of circulating hCD45 immune cells (78 vs. 63% on average for the others), which may have contributed to the increased growth. Surprisingly, when hiPSCs were injected in Hu-BLT mice reconstituted with allogeneic immune cells, three out six teratomas were not totally rejected (Figure 3B). However, the fact that these three remaining teratomas were massively infiltrated by T cells and undergoing high levels of apoptosis suggests that they would have been eventually eliminated as observed when using allogeneic Hu-AT mice (Figure 3C).

### Natural Killer Cells Fail to Reject Established Teratomas

**Figure 4:**
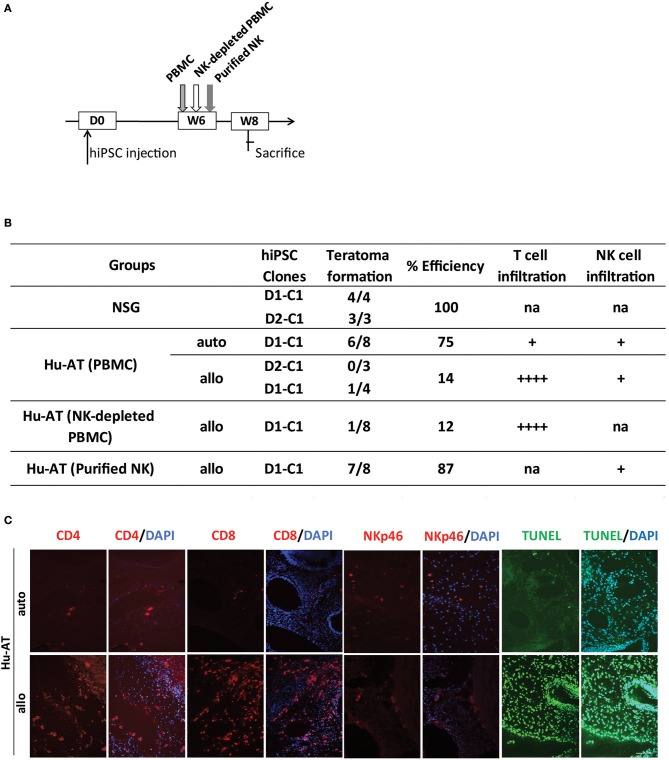
Established teratomas are not the target of NK cells in Hu-AT mice. **(A)** Experimental design and timeline of the injection of hiPSCs in Hu-AT mice. Approximately 1 × 10^6^ hiPSCs were first injected under the renal capsule of NSG mice. Six weeks later (W6), mice were injected intravenously with PBMCs, NK-depleted PBMCs, or purified NK cells. Two weeks after the adoptive transfer of immune cells (W8), mice were sacrificed, and the incidence of teratomas and the infiltration of immune cells were determined. **(B)** Incidence of teratoma formation in the renal capsule of Hu-AT mice. Shown is the proportion of teratomas derived from the indicated hiPSC clones and their relative infiltration by T and NK cells. Overall, a significant difference was observed between Hu-AT (NK-depleted PBMC) allogeneic and Hu-AT (purified NK) allogeneic, χ^2^(4) = 21.15, *p* < 0.05. na indicates not applicable, + indicates a low level of infiltration (<10 cells/field on average), and ++++ indicates a high level of infiltration (more than 100 cells/field on average). **(C)** Representative photos showing the infiltration of CD4^+^, CD8^+^ T cells, and NKp46^+^ NK cells (in red) and apoptosis (as determined by TUNEL in green) in sections of teratomas collected from Hu-AT mice injected with allogeneic or autologous 1 × 10^7^ PBMCs. Of note, only one teratoma was retrieved in mice injected with allogeneic PBMCs and used for the staining. DAPI staining was performed to visualize nuclei (in blue). Showed are photos taken at 20×. Scale bar, 100 μm. NK, natural killer; hiPSCs, human induced pluripotent stem cells; NSG, NOD/SCID/IL2Rγ^null^; PBMCs, peripheral blood mononuclear cell.

We then hypothesized that the protection against the formation of teratomas conferred by NK cells was attributed to the undifferentiated nature of iPSCs (i.e., low HLA-I expression as shown in Figure S1C), which is known to favor the activation of NK cells. To test this hypothesis, we repeated the above-described experiment, but this time, we proceeded to the AT of immune cells only 6 weeks after the injection of hiPSCs, a time at which teratomas had already started to grow and cells differentiate (Figure 4A). Although we found that allogeneic PBMCs or NK-depleted PBMCs were capable of rejecting teratomas (1/7 and 1/8, respectively), autologous PBMCs, and purified NK cells were not (6/8 and 7/8, respectively, see Figure 4B). Overall, a significant difference was observed between the different conditions, χ^2^(4) = 21.15, *p* < 0.05. Further analysis reveals a significant difference between Hu-AT (NK-depleted PBMC) allogeneic and Hu-AT (purified NK) allogeneic (*p* < 0.05). No other significant difference was detected. Analysis of the teratomas upon sacrifice showed a massive T-cell infiltration and apoptosis in the remaining allogeneic teratomas (Figure 4C). In comparison, only a few T cells were found in autologous teratomas. As expected, very few NK cells were found in growing teratomas, suggesting that these cells lose their ability to recognize differentiated hiPSC-derived cells. This is supported by the fact that while hiPSCs expressed very low levels of HLA-I expression, these levels were found much higher within only a few hours after the cells were placed in the presence of serum *in vitro* (Figure S4). Overall, these results indicate that NK cells are very effective at preventing the formation of a teratoma from hiPSCs but lose this capacity against differentiated cells.

## Discussion

The ability to generate hiPSCs at an affordable cost and in a timely manner will be necessary to assure the success of future hiPSC-derived cell therapies. Moreover, it will be essential to demonstrate that hiPSC-based therapies, whether using autologous donors or through the development of universal cell lines, are safe (13–15). Using two distinct humanized mouse models, we here provide evidences that hiPSCs are the target of NK cells *in vitro* and *in vivo*. In contrast to what was found using murine iPSCs, human NK cells could prevent the formation of teratomas without prior activation (7). Other than the species differences, such disparity may be explained by the use of a different injection site in our study (renal capsule vs. subcutaneous) and perhaps by the high expression level of NK activating ligands (i.e., MICA/B) in hiPSCs. Another explanation may be that immune cells, including NK cells, may have been already partially or fully activated from their injection into NSG mice, which are known to gradually develop severe graft-vs.-host disease (GvHD) following the injection of human PBMCs. Our results also showed that in the absence of NK cells, teratomas from allogeneic but not autologous hiPSCs were rejected by T cells in both Hu-AT and Hu-BLT mice. Moreover, we found that inhibition of teratoma formation was possible only if hiPSCs were injected prior to the AT of total PBMCs or purified NK cells. In addition, in the absence of recombinant human IL-15 to support the proliferation of NK in our mice (16), our results also suggest that the relatively low number of NK cells injected during the AT procedure was sufficient to prevent the growth of teratomas. These results suggest that if immunosuppressive drugs were to be used to increase engraftment of iPSC-derived cells, it would be very important to insure no inhibition of the NK cell compartment to lower the risk of forming a teratoma.

## Data Availability Statement

The datasets generated for this study are available on request to the corresponding author.

## Ethics Statement

The animal study was reviewed and approved by CHU Sainte-Justine comité d'éthique de la recherche.

## Author Contributions

BB, CD-L, CC, and YL performed experiments. GR performed statistical analysis. BB, EH, and CB designed the studies. JG provided cells. BB and CB wrote the manuscript.

### Conflict of Interest

The authors declare that the research was conducted in the absence of any commercial or financial relationships that could be construed as a potential conflict of interest.
