# Supplementary material for: Natural Killer Cells Prevent the Formation of Teratomas Derived From Human Induced Pluripotent Stem Cells
Source: Front Immunol. 2019 Nov 7;10:2580. doi: 10.3389/fimmu.2019.02580 (PMC6854018; doi:10.3389/fimmu.2019.02580)
Supplement: Supplementary file 1 [file Presentation_1.pptx]

## Slide 1
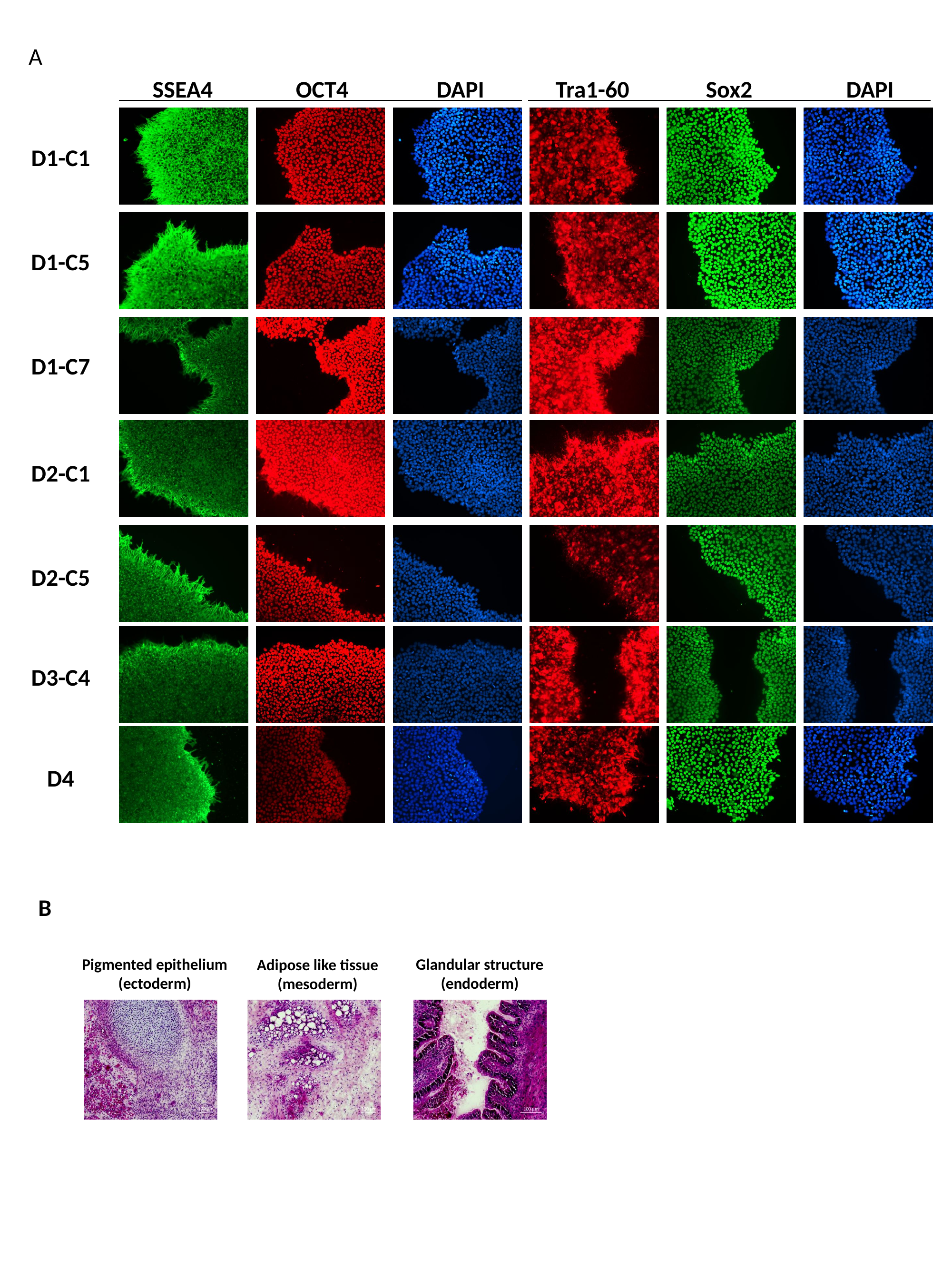

A
SSEA4
OCT4
DAPI
Tra1-60
Sox2
DAPI
D1-C1
D1-C5
D1-C7
D2-C1
D2-C5
D3-C4
D4
B
Pigmented epithelium
(ectoderm)
Glandular structure
(endoderm)
Adipose like tissue
(mesoderm)

## Slide 2
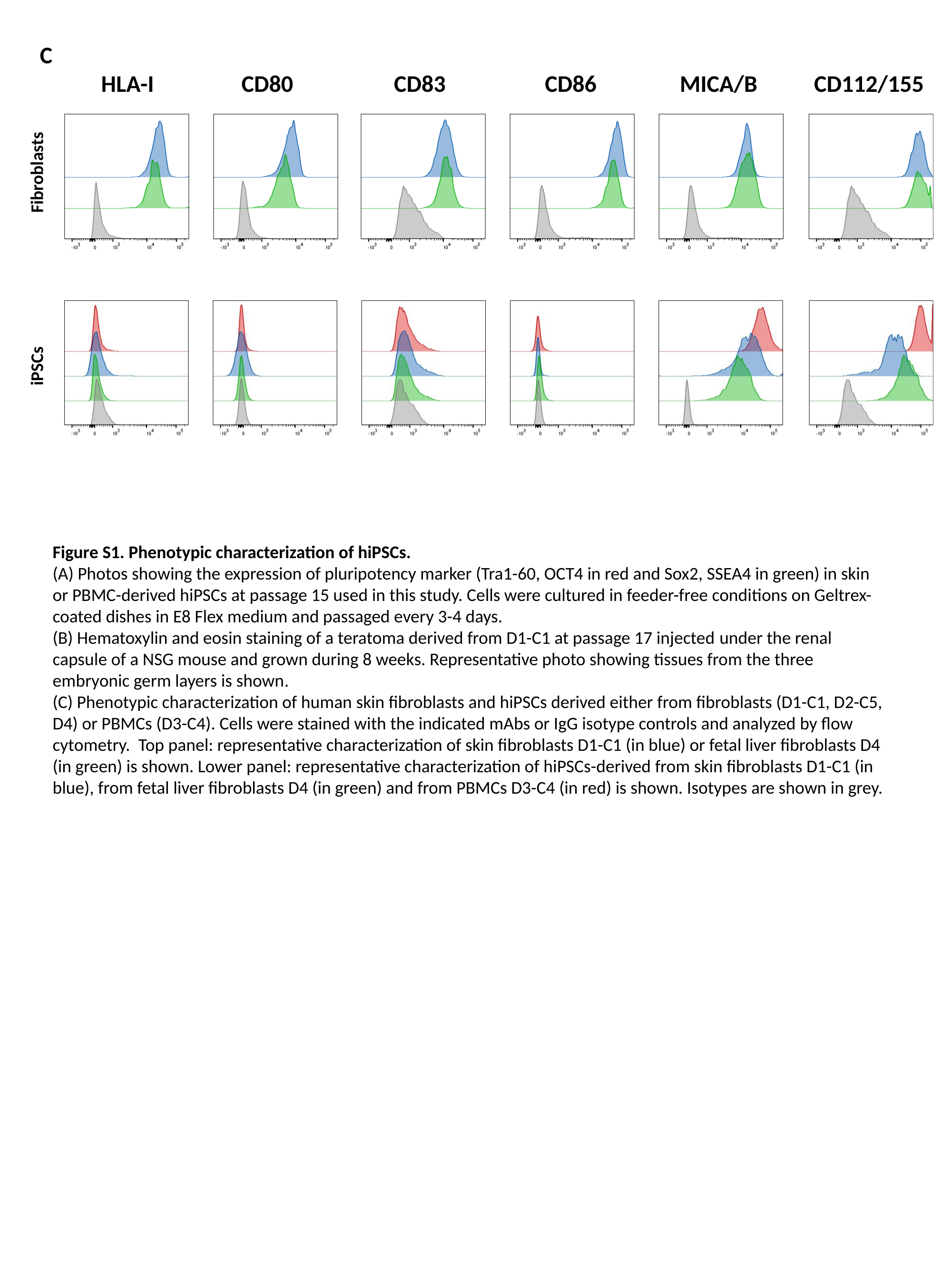

C
HLA-I
CD80
CD83
CD86
MICA/B
CD112/155
Fibroblasts
iPSCs
Figure S1. Phenotypic characterization of hiPSCs.
(A) Photos showing the expression of pluripotency marker (Tra1-60, OCT4 in red and Sox2, SSEA4 in green) in skin or PBMC-derived hiPSCs at passage 15 used in this study. Cells were cultured in feeder-free conditions on Geltrex-coated dishes in E8 Flex medium and passaged every 3-4 days.
(B) Hematoxylin and eosin staining of a teratoma derived from D1-C1 at passage 17 injected under the renal capsule of a NSG mouse and grown during 8 weeks. Representative photo showing tissues from the three embryonic germ layers is shown.
(C) Phenotypic characterization of human skin fibroblasts and hiPSCs derived either from fibroblasts (D1-C1, D2-C5, D4) or PBMCs (D3-C4). Cells were stained with the indicated mAbs or IgG isotype controls and analyzed by flow cytometry. Top panel: representative characterization of skin fibroblasts D1-C1 (in blue) or fetal liver fibroblasts D4 (in green) is shown. Lower panel: representative characterization of hiPSCs-derived from skin fibroblasts D1-C1 (in blue), from fetal liver fibroblasts D4 (in green) and from PBMCs D3-C4 (in red) is shown. Isotypes are shown in grey.

## Slide 3
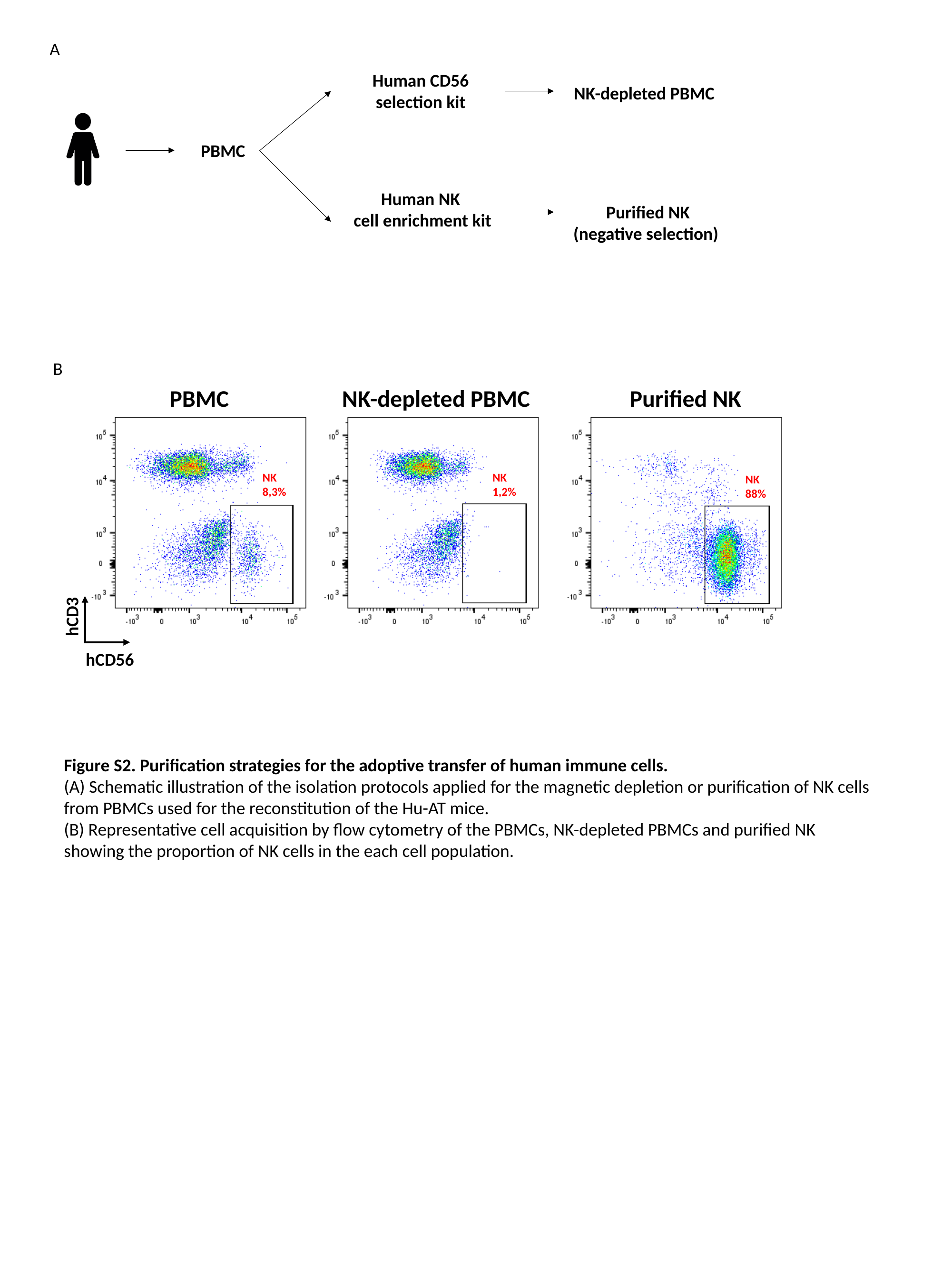

A
Human CD56 selection kit
NK-depleted PBMC
PBMC
Human NK
cell enrichment kit
Purified NK
(negative selection)
B
PBMC
NK-depleted PBMC
Purified NK
NK
8,3%
NK
1,2%
NK
88%
hCD3
hCD56
Figure S2. Purification strategies for the adoptive transfer of human immune cells.
(A) Schematic illustration of the isolation protocols applied for the magnetic depletion or purification of NK cells from PBMCs used for the reconstitution of the Hu-AT mice.
(B) Representative cell acquisition by flow cytometry of the PBMCs, NK-depleted PBMCs and purified NK showing the proportion of NK cells in the each cell population.

## Slide 4
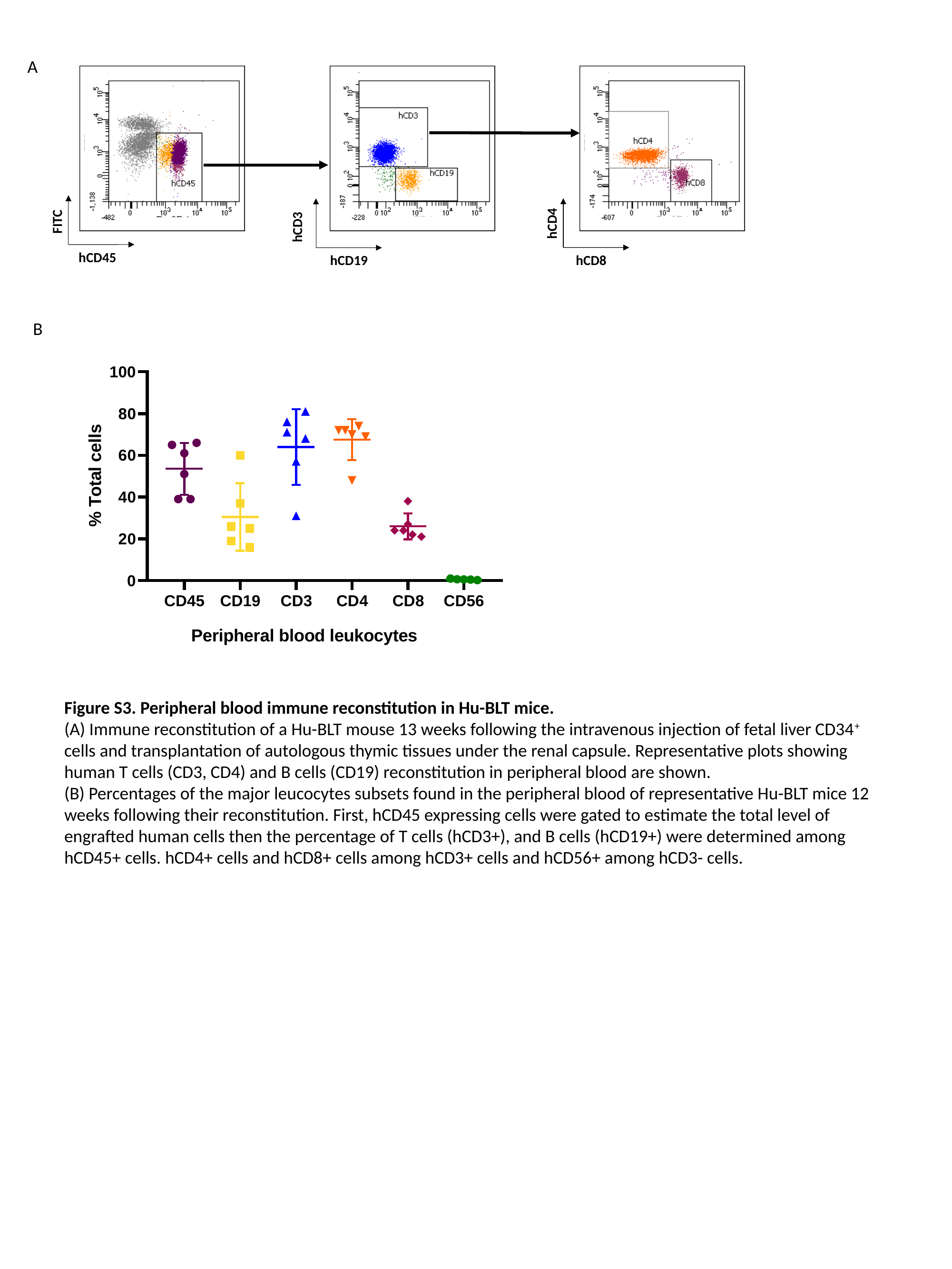

A
FITC
hCD4
hCD3
hCD45
hCD8
hCD19
B
Figure S3. Peripheral blood immune reconstitution in Hu-BLT mice.
(A) Immune reconstitution of a Hu-BLT mouse 13 weeks following the intravenous injection of fetal liver CD34+ cells and transplantation of autologous thymic tissues under the renal capsule. Representative plots showing human T cells (CD3, CD4) and B cells (CD19) reconstitution in peripheral blood are shown.
(B) Percentages of the major leucocytes subsets found in the peripheral blood of representative Hu-BLT mice 12 weeks following their reconstitution. First, hCD45 expressing cells were gated to estimate the total level of engrafted human cells then the percentage of T cells (hCD3+), and B cells (hCD19+) were determined among hCD45+ cells. hCD4+ cells and hCD8+ cells among hCD3+ cells and hCD56+ among hCD3- cells.

## Slide 5
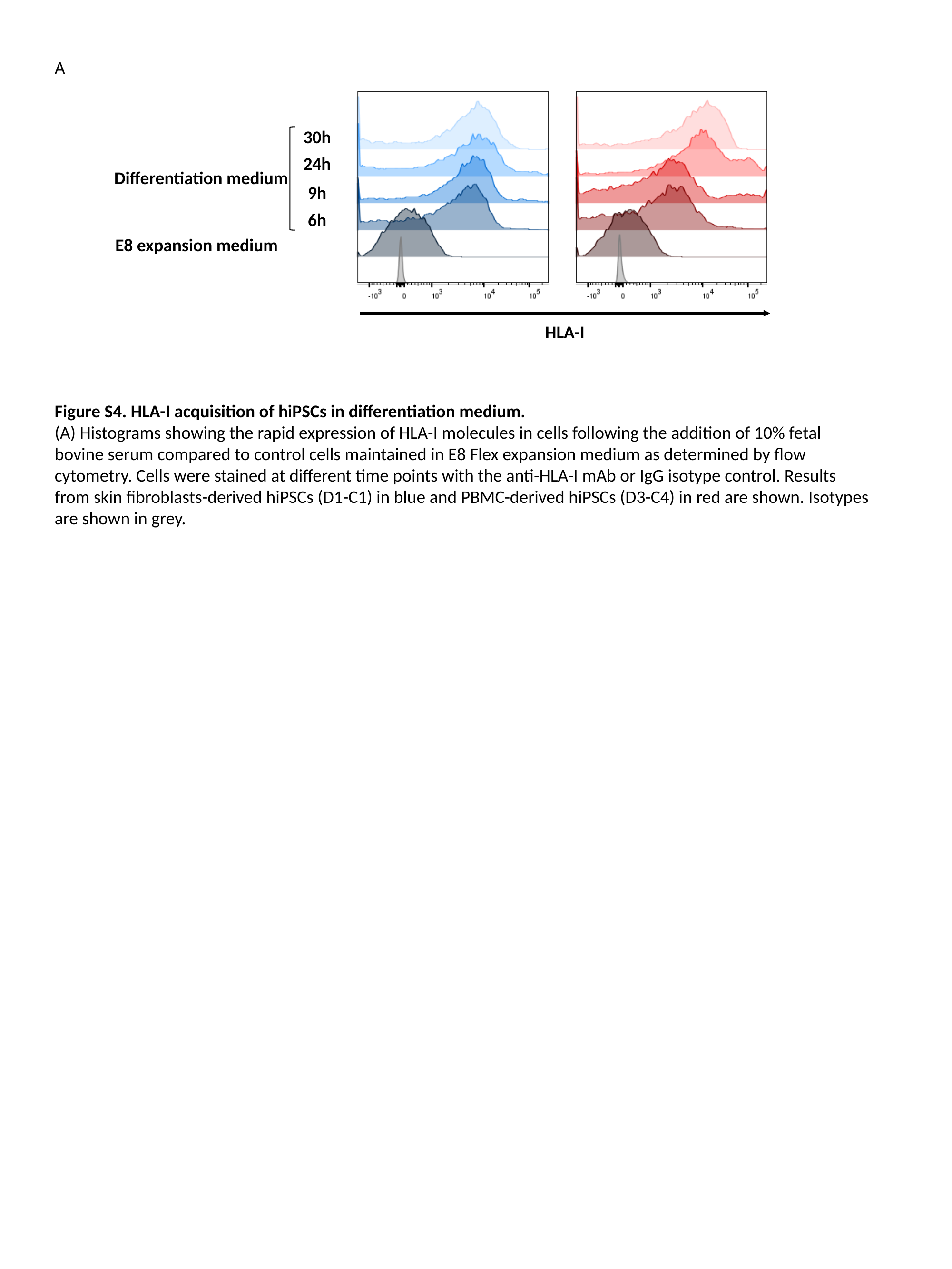

A
30h
24h
Differentiation medium
9h
6h
E8 expansion medium
HLA-I
Figure S4. HLA-I acquisition of hiPSCs in differentiation medium.
(A) Histograms showing the rapid expression of HLA-I molecules in cells following the addition of 10% fetal bovine serum compared to control cells maintained in E8 Flex expansion medium as determined by flow cytometry. Cells were stained at different time points with the anti-HLA-I mAb or IgG isotype control. Results from skin fibroblasts-derived hiPSCs (D1-C1) in blue and PBMC-derived hiPSCs (D3-C4) in red are shown. Isotypes are shown in grey.

## Slide 6
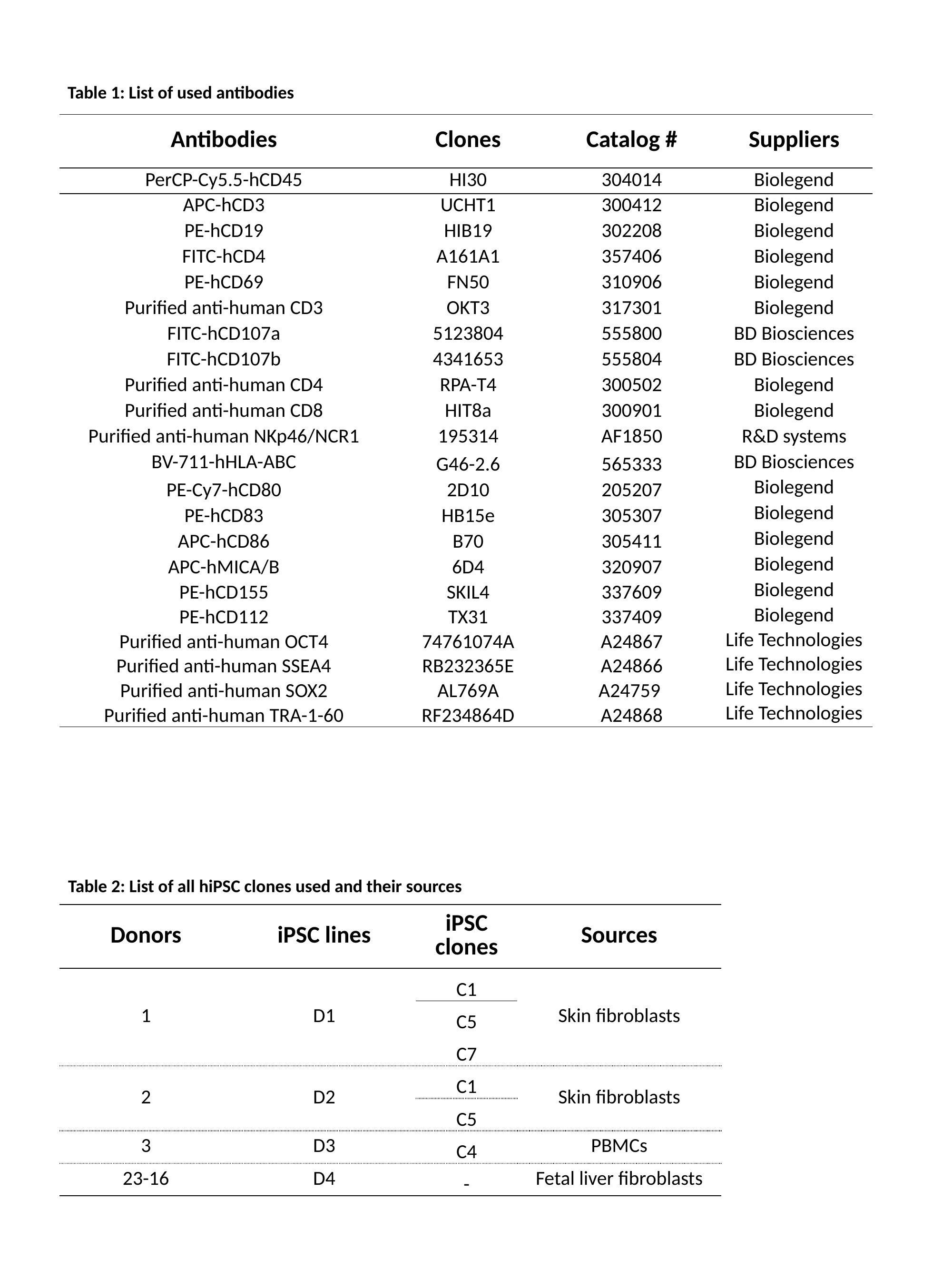

Table 1: List of used antibodies
| Antibodies | Clones | Catalog # | Suppliers |
| --- | --- | --- | --- |
| PerCP-Cy5.5-hCD45 | HI30 | 304014 | Biolegend |
| APC-hCD3 | UCHT1 | 300412 | Biolegend |
| PE-hCD19 | HIB19 | 302208 | Biolegend |
| FITC-hCD4 | A161A1 | 357406 | Biolegend |
| PE-hCD69 | FN50 | 310906 | Biolegend |
| Purified anti-human CD3 | OKT3 | 317301 | Biolegend |
| FITC-hCD107a | 5123804 | 555800 | BD Biosciences |
| FITC-hCD107b | 4341653 | 555804 | BD Biosciences |
| Purified anti-human CD4 | RPA-T4 | 300502 | Biolegend |
| Purified anti-human CD8 | HIT8a | 300901 | Biolegend |
| Purified anti-human NKp46/NCR1 | 195314 | AF1850 | R&D systems |
| BV-711-hHLA-ABC | G46-2.6 | 565333 | BD Biosciences |
| PE-Cy7-hCD80 | 2D10 | 205207 | Biolegend |
| PE-hCD83 | HB15e | 305307 | Biolegend |
| APC-hCD86 | B70 | 305411 | Biolegend |
| APC-hMICA/B | 6D4 | 320907 | Biolegend |
| PE-hCD155 | SKIL4 | 337609 | Biolegend |
| PE-hCD112 | TX31 | 337409 | Biolegend |
| Purified anti-human OCT4 | 74761074A | A24867 | Life Technologies |
| Purified anti-human SSEA4 | RB232365E | A24866 | Life Technologies |
| Purified anti-human SOX2 | AL769A | A24759 | Life Technologies |
| Purified anti-human TRA-1-60 | RF234864D | A24868 | Life Technologies |
Table 2: List of all hiPSC clones used and their sources
| Donors | iPSC lines | iPSC clones | Sources |
| --- | --- | --- | --- |
| 1 | D1 | C1 | Skin fibroblasts |
| | | C5 | |
| | | C7 | |
| 2 | D2 | C1 | Skin fibroblasts |
| | | C5 | |
| 3 | D3 | C4 | PBMCs |
| 23-16 | D4 | - | Fetal liver fibroblasts |
